# Lead Assays with Smartphone Detection Using a Monolithic Rod with 4-(2-Pyridylazo) Resorcinol

**DOI:** 10.3390/molecules26185720

**Published:** 2021-09-21

**Authors:** Piyanat Issarangkura Na Ayutthaya, Chonnipa Yeerum, Kullapon Kesonkan, Kanokwan Kiwfo, Kate Grudpan, Norio Teshima, Hiroya Murakami, Monnapat Vongboot

**Affiliations:** 1 Department of Chemistry, Faculty of Science, King Mongkut’s University of Technology Thonburi, Bangkok 10140, Thailand; piyanat.tp@gmail.com (P.I.N.A.); chonnipa.yeerum@gmail.com (C.Y.); kullapon.kesonkan@gmail.com (K.K.); 2 Center of Excellence for Innovation in Analytical Science and Technology and Department of Chemistry, Faculty of Sciences, Chiang Mai University, Chiang Mai 50200, Thailand; k.kanokwan11@gmail.com; 3 Department of Applied Chemistry, Aichi Institute of Technology, 1247 Yachigusa, Yakusa-cho, Toyota 470-0392, Japan; teshima@aitech.ac.jp (N.T.); hmurakami@aitech.ac.jp (H.M.)

**Keywords:** monolithic polyurethane foam–[4-(2-pyridylazo) resorcinol], chemical sensor, lead, smartphone, on-site screening, water monitoring

## Abstract

A monolithic rod of polyurethane foam–[4-(2-pyridylazo) resorcinol] (PUF–PAR) as a simple chemical sensor for lead assays with smartphone detection and image processing was developed. With readily available simple apparatus such as a plastic cup and a stirrer rod, the monolithic PUF rod was synthesized in a glass tube. The monolithic PUF–PAR rod could be directly loaded by standard/sample solution without sample preparation. A one-shot image in G/B value from a profile plot in ImageJ for a sample with triplicate results via a single standard calibration approach was obtained. A linear single standard calibration was: [G/B value] = −0.038[µg Pb^2+^] + 2.827, R^2^ = 0.95 for 10–30 µg Pb^2+^ with a limit of quantitation (LOQ) of 33 µg L^−1^. The precision was lower than 15% RSD. The proposed method was tested by an assay for Pb^2+^ contents in drinking water samples from Bangkok. The results obtained by the proposed method agree with those of ICP-OES and with 100–120% recovery, demonstrating that the method is useful for screening on-site water monitoring.

## 1. Introduction

Lead is a substance of concern due to its toxicity. According to Thailand’s guidelines for health effects, a maximum of 50 µg L^−1^ of lead is allowed in drinking water [1]. The standard methods for water analysis usually employ colorimetry (using dithizone reagent), atomic absorption spectroscopy (AAS) with flame and non-flame, inductively coupled plasma optical emission spectrometry/mass spectrometry (ICP-OES/MS), and anodic stripping voltammetry (ASV) [2]. The development of lead determination has been of interest for various kinds of applications, including water monitoring. Even in recent decades, there have been a number of reports devoted to lead monitoring, including the use of nanoparticles [3,4,5,6] and smartphones [7,8,9,10,11], although various screen-printed electrodes have been used for the electrochemical analysis of lead [12,13,14], with the aims of improved sensitivity and more convenient procedures.

4-(2-pyridylazo) resorcinol (PAR) has been a color reagent of interest for lead determination since the 1960s [15], due to its good solubility in water, rapid color formation, reasonable sensitivity, and high stability for lead complexes [16]. PAR, which could be viable in laboratories, has made the colorimetric determination of lead a popular implementation, with simple operation. Pretreatment may be associated with the colorimetric determination of lead using PAR. Some sorbents such as AV-17 [17,18,19], polyurethane foam (PUF) [20], Amberlite XAD-1180 [21], Amberlite XAD-7 [22], modified silica [23], imprinted polymer nanoparticles [24], modified nano-alumina [6], nanomagnetic materials [3,5], TrisKem Pb resin [25], NOBIAS chelate PA-1 [26], Amberlite IR-120 [27], PB-resin [28], and nonwoven polypropylene [29] have been employed. Some of those were used to sorb lead with elution, for the next step of forming color with PAR for a colorimetric assay [22,25,26,27,28]. In some previous works, lead that formed anionic complexes before being sorbed on AV-17 could then form a color complex when treated with a PAR solution [17,18,19]. The silica was treated with mixed ligands for the sorption of lead before forming a color product with PAR [23]. In both the latter cases, detection with diffuse reflectance spectrometry was employed.

Polyurethane foam (PUF) has gained interest for use as a sorbent for lead [20,30,31,32,33,34,35,36,37,38,39,40,41,42,43,44,45,46,47]. Many previous works have been devoted to pretreatment for the determination of lead by atomic absorption spectrometry. Only one of them was applied using PAR [20]; PUF, as purchased, was made into a powder and packed into a column, then loaded with PAR in a flow injection system. The eluted lead from the column was then allowed to flow into AAS for lead determination.

Recently, our research group introduced PUF as a monolithic rod with the single standard calibration approach for anionic surfactant assays, employing methylene blue reagent [48] and a PUF–alginate monolithic rod for lead determination using flow injection–flame atomic absorption spectrometry [49].

It would, therefore, be of interest to make use of PUF loading with PAR as a monolithic column to sorb lead, producing a color product (Pb^2+^–PAR sorbed on PUF) with the use of smartphone detection for various expected benefits, such as simplicity in monolithic PUF–PAR rod fabrication for a ready-to-use chemical sensor, according to the IUPAC definition of a sensor [50] with a one-shot image, and the advantage of the single standard calibration approach without sample preparation for on-site water monitoring.

## 2. Results

### 2.1. The Monolithic PUF–PAR Rod

**Figure 1:**
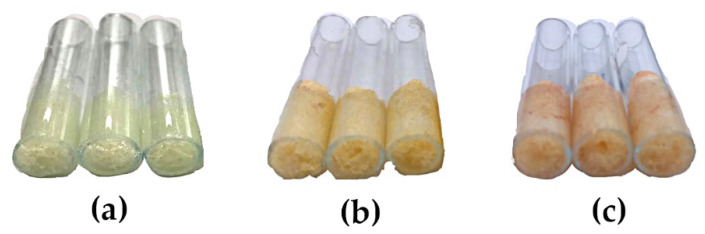
Monolithic rods of: (**a**) PUF; (**b**) PUF–PAR; and (**c**) PUF–PAR after passing a Pb^2+^ solution.

A monolithic PUF rod (obtained from the synthesis) of a cylindrical shape, 2 cm in height, with good porosity characteristics, was instantly packed into the glass rod to create a mold during the synthesis step. The color of the obtained monolithic PUF rod was white (see Figure 1a). After loading with a PAR solution, and being left to dry, the PUF immobilized with PAR resulted in a yellow monolithic PUF–PAR rod, as depicted in Figure 1b. When passing a Pb^2+^ solution, red coloration appeared on the monolithic PUF–PAR rod (see Figure 1c). The higher the concentration of Pb^2+^, the more intense the color observed. In one batch of fabrication of the monolithic PUF–PAR rod, i.e., synthesizing the backbone monolithic PUF rod in a glass rod as a mold, and immobilizing PAR, 40 monolithic rods could be obtained, and were ready to use as working monolithic PUF–PAR rods for lead assays (cf. A in Figure S1). The working monolithic PUF–PAR rod could be kept in a desiccator for further use for at least a week.

### 2.2. The Proposed Water Monitoring Procedure

**Figure 2:**
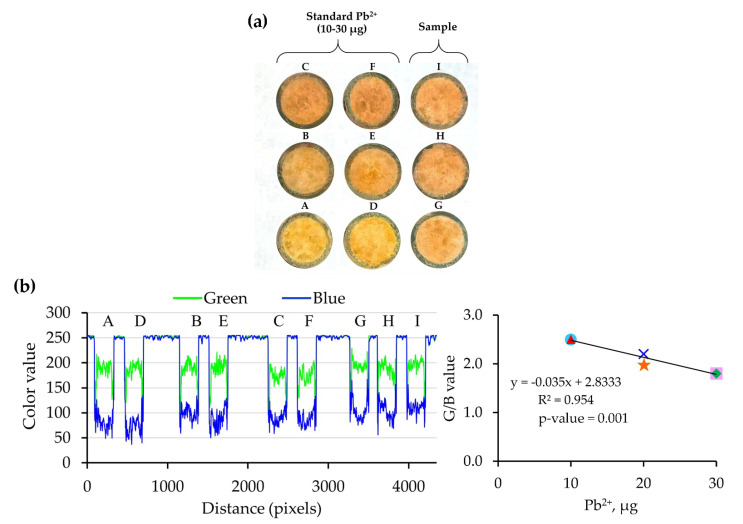
Assay using the proposed procedure for one-shot image of a sample with triplicate results via a single standard calibration approach: (**a**) one-shot photo taken of the nine rods (A−F due to the Pb^2+^ standards, G–I due to a sample with different loading volumes); (**b**) profile of G and B values with calibration graph (see Table 1): A (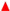), B (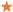), C (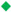), D (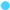), E (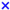), F (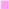).

An analyte solution (standard/sample) with the desired volume was loaded through a monolithic PUF–PAR rod and left to dry. A set of nine rods (six rods for Pb^2+^ standards, and three rods of a sample for triplicate results) was employed for each sample (see Figure 2). The one-shot image was obtained using a smartphone under the light control box. Image processing was carried out using ImageJ for the ratio of the G/B value, where the G value and B value refer to the color intensities of the color mode: red (R), green (G), and blue (B).

It is noted that the above analysis procedure is composed of two steps: detection (loading and photographing) and evaluation (cf. B and C in Figure S1). After loading, it was left to dry (for approximately two hours) before photographing. During the drying period, operation of the other sites could be performed in parallel. The photographing would take less than one minute, and then it would take less than five minutes for evaluation, including image processing, to obtain the triplicate analysis results. This is still useful for the on-site monitoring approach.

Loaded Pb^2+^ (in microgram) on a monolithic PUF–PAR rod could be computed by: Pb^2+^ microgram = C_Pb_^2+^ × FR_analyte_ _solution_ × LT, where C_Pb_^2+^ is the Pb^2+^ concentration (µg mL^−1^) in the solution, FR_analyte_ _solution_ is the flow rate of analyte (mL min^−1^), and LT is the loading time (min). A linear calibration graph can be established by a plot of the Pb^2+^ microgram against the G/B value. However, the equation can be reduced to the shorter form: Pb^2+^ microgram = C_Pb_^2+^ × V_analyte_ _solution_, where V_analyte_ _solution_ is the loading volume.

A linear calibration graph, using a single standard calibration approach (see detail in Section 3.3), was obtained by a plot of the Pb^2+^ microgram against the G/B value (see Figure 2).

The calibration equation was: G/B value = −0.038[µg Pb^2+^] + 2.827 (R^2^ = 0.95) for the linear range of 10–30 µg Pb^2+^. The precision of the proposed method was less than 15% RSD.

It can be observed from Table 1 that using a solution of 0.4 µg mL^−1^ Pb^2+^ for I–G positions, with different loading volumes, resulted in different µg from the single standard calibration but yielded the same concentration values. In this way, triplicate results could be obtained even when using different loading volumes. This offers a method through which to verify the obtained results.

**Figure 3:**
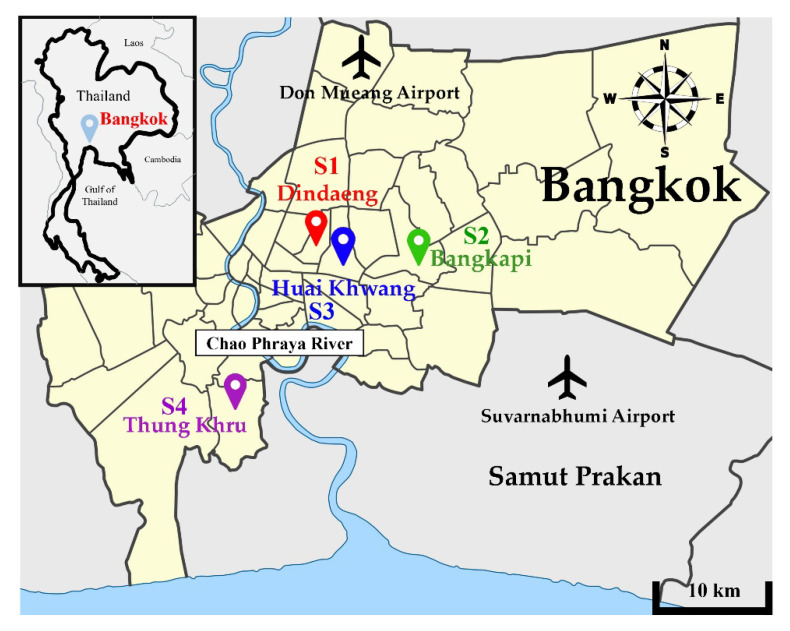
Sampling areas in Bangkok, Thailand for lead assays of drinking water samples.

The developed procedure was tested for real application in assays of Pb^2+^ in drinking water samples from coin-operated drinking water vending machines in various districts of Bangkok (see Figure 3), Thailand, including Dindaeng (S1), Bangkapi (S2), Huai Khwang (S3), and Thungkru (S4). The pH values of the drinking water samples were found to be 7 ± 0.5, so the samples were directly loaded to the monolithic PUF–PAR rod without any sample pretreatment. The results are summarized in Table 2.

The results obtained by the proposed method agree with those obtained by the ICP-OES method. When spiking each sample with 50 Pb^2+^ std (µg L^−1^), which is the maximum acceptable concentration of lead in drinking water, the results obtained by the proposed procedure are 50 ± 10 µg L^−1^, indicating that the sensitivity of the proposed procedure is appropriate for screening water in accordance with Thailand’s guidelines for the maximum allowance of lead [1].

It was found that the observed values were less than the theoretical values (2.92 at 95% confidence level), indicating no significant difference. The aim of this work is to report the development of simple procedures for lead assays employed in water monitoring. However, we are aware that the number of samples needs to be increased to verify the utility of the method. Further work regarding water monitoring on a larger scale is planned.

## 3. Discussion

### 3.1. Properties of the Monolithic PUF–PAR Rod

Unlike that mentioned earlier (that PUF used in previous works was commercially available), in this work, the monolithic PUF rod was synthesized by modifying the work of [48,49] and by utilizing readily available simple apparatus such as a plastic cup and a stirrer rod. Methylene diphenyl diisocyanate (MDI) was mixed for a few minutes with polyol and additives (water and silicone oil). By plugging the glass rods into the mixture, polymerization reactions took place within five minutes in the glass rods, which served as molds within which to form the monolithic rods. PAR was immobilized onto the PUF monolithic rods by loading with PAR solution for one hour and being left to dry overnight.

Employing the 2:1 ratio of diisocyanate groups from MDI and polyol groups could properly generate rigid PUF in the rod due to the potential rigidity of the aromatic parts provided by MDI. The white foam could be achieved from this ratio, as there is no profusion of brown color from MDI, resulting in no brown color interfering in the colorimetric detection step. PUF is shrunken and cohesive when the ratio of the polyol is higher than that of MDI, on account of the excess softened part for forming the monolithic PUF rod. Water, as a chemical blowing agent, serves to enlarge the porous cell and enhance the height of the foam by the generation of ${\mathrm{CO}}_{2}$ during the polymerization processes. Silicone oil, as a surfactant, serves to control the size of the porous cell, and increase the numbers of open cells. Consequently, this enhances the porosity characteristics (higher number of pores, higher surface area, and well-controlled porousness), resulting in the smooth flow of the solution and the whiteness of the obtained PUF. The obtained monolithic PUF rod possesses these properties, while the PUF in powder lacks them.

The resulting characteristics of the obtained PUF monolithic rods offer advantages over the PUF in powder form that was used in previous works [20,30,31,34,35,36,38,40,42,45,51].

**Figure 4:**
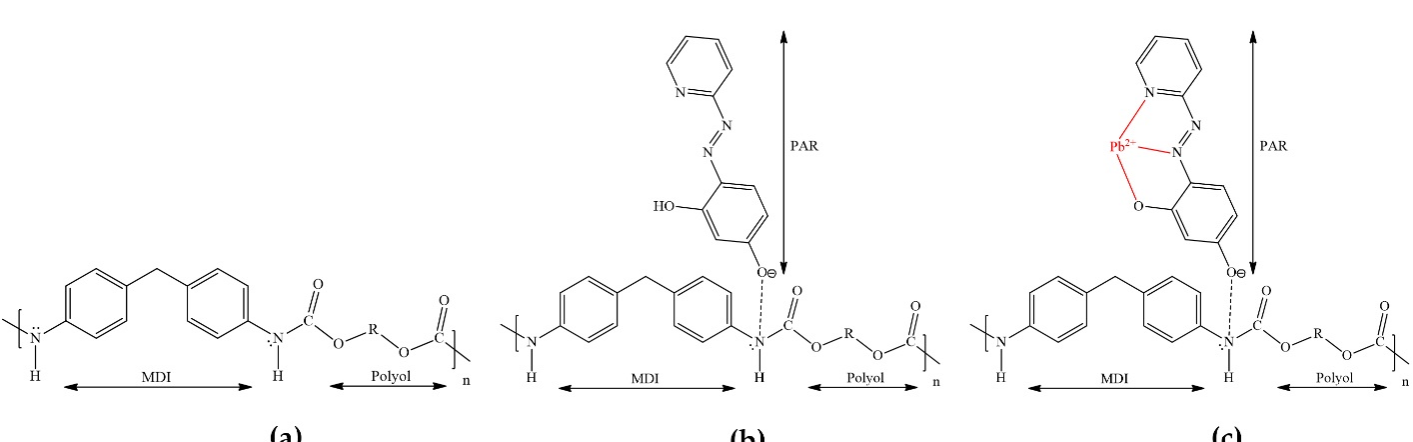
Proposed chemical structures of: (**a**) PUF; (**b**) PUF–PAR; and (**c**) PUF–PAR after passing a Pb^2+^ solution.

The chemical structure of PUF consists of two parts: the diisocyanate group (-NHCO-) and the polyol groups (-C-O-C-), which are depicted in Figure 4a, relating to Figure 1a. It could be that the monolithic PUF performs as a weak positive charge [52] due to the inducing of aromatic rings and carbonyl groups, as shown in Figure 4b in connection to Figure 1b. At pH 7, PAR as the negative charge (denoted as HPAR¯) may sorb on an amino group of PUF [53]. Typically, toluene diisocyanate (TDI), well known for its isocyanate groups, is used in commercialized PUFs. Using the diisocyanate group from MDI instead of TDI can possibly support the amino group of PUF to be the more positive charge, because MDI includes two aromatic rings, whereas TDI has only one. For this reason, the amino group of the urethane bond may enhance the transference of electrons to the aromatic ring because of its electron-donating character. Moreover, the highly positive charge of the amino group predominant in commercialized PUFs and the sorption of PAR could be strong. After passing the Pb^2+^ solution, it would sorb on the monolithic PUF–PAR rod as Pb^2+^–PAR complexes. Pb^2+^ may bind with the pyridine nitrogen atom, azo-nitrogen atom, and o-hydroxyl group of PAR (see Figure 4c, associated with Figure 1c) [53].

A flow rate of 13 ± 5 mL min^−1^ was observed for gravitational flow through monolithic PUF–PAR rods (*n* = 90), so the procedure was designed without using a pump. A sample can be directly loaded into the monolithic PUF–PAR rod without any extra sample preparation.

### 3.2. Parameters Affecting the Color Development: PAR Concentration and pH

In the preliminary investigation, color measurement, PUF–PAR in powder form was studied by adding the PUF–PAR powder (0.25 g) to Pb^2+^ solutions (0.1–100 µg mL^−1^). The mixture was shaken by a horizontal shaker at 110 rpm for two hours. The powder was filtered out and dried before taking a photo with a smartphone camera under the light control box. It was observed that the color of the PUF–PAR powder changed from yellow to red in accordance with the PAR and the Pb^2+^–PAR complex formation. The image characteristics were studied. It was found that the R (red), G (green), and B (blue) values, which are the color properties, did not have any direct correlation with the Pb^2+^ concentration. The CMYK (cyan, magenta, yellow, key) mode provided no direct correlation either. It was found that G/B (green divided by blue) or R/B (red divided by blue) values resulted in a linear relationship with the Pb^2+^ concentration; the ratio of the G/B value was chosen for further study, as the G/B value resulted in higher sensitivity.

#### 3.2.1. PAR Concentrations

Instead of a column packed with PUF–PAR powder, a monolithic PUF–PAR rod was used, as it offers smooth passage through solution. The concentration of PAR was an important parameter for the color development of Pb^2+^–PAR complexes in which an excess amount of PAR was required. The effect of PAR concentration was studied for PAR concentrations at 0.01, 0.05, and 0.1% *w*/*v*. The concentrations of Pb^2+^ varying from 0.5 to 5 µg mL^−1^ were percolated into the monolithic PUF–PAR rod with a certain volume. No significant difference was observed for coloration of Pb^2+^–PAR complexes via the G/B value of 0.05 and 0.1% *w*/*v* PAR, while 0.01% *w*/*v* PAR produced lower signals of G/B value, indicating an insufficient amount of the reagent for complex formation. The concentration of 0.05% *w*/*v* PAR was chosen for further study.

#### 3.2.2. Effect of pH

**Figure 5:**
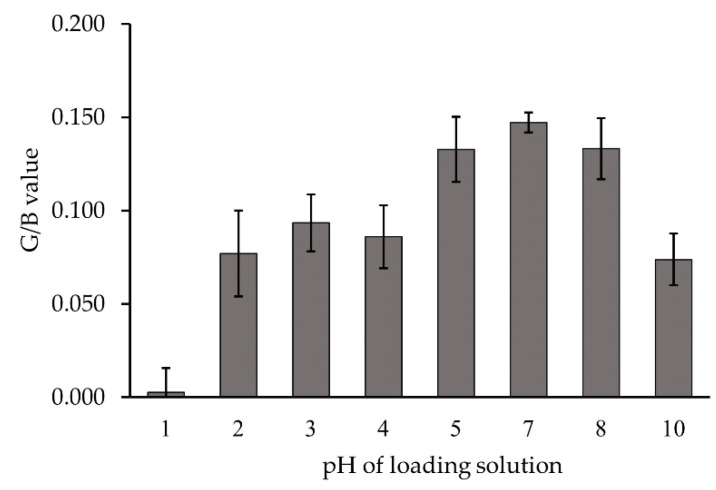
Effect due to pH of loading solution (see text in Table 1).

PAR consists of four diverse forms that depend on the pH of the solution, and persist with different charges and colors, namely $H_{3}L^{+}\;$(yellow), $H_{2}L$ (yellow-orange), ${\mathrm{HL}}^{-}$ (orange), and $L^{2-}$ (red) [53]. The wavelength of the maximum absorption of the red Pb^2+^–PAR complex is 520 nm at various pH, ranging from pH 4 to 12 [15]. As the Pb^2+^–PAR complexes were formed on the PUF–PAR surface, the pH of the loading solution affected the form of PAR on the surface. Therefore, the optimal pH of the loading solution was then investigated. Each of the solutions, containing 20 µg Pb^2+^ with pH varying from 1 to 10, was percolated to the monolithic PUF–PAR rod. The effect of pH on the G/B value, and thus on the amount of the Pb^2+^–PAR complexes, is represented in Figure 5. Pb^2+^–PAR complexes rarely formed at pH 1 and partially formed at pH 2−4, because hydrogen ions competitively protonate the nitrogen atom of the pyridine ring of PAR [53]. On the other hand, in the basic medium (pH 8–10), the complexation of the Pb^2+^–PAR complex was decreased because of the plausible interaction between Pb^2+^ and hydroxide ions such as ${\mathrm{Pb}(\mathrm{OH})}_{2}\;$at pH 10, so the Pb^2+^–PAR complexes could scarcely occur [54]. Obviously, pH 7 provided the maximum G/B value because of the existent forms of $H_{2}L$ and ${\mathrm{HL}}^{-}$ of PAR. The occurrence of Pb^2+^–PAR complexes on PUF at pH 7 was found to be appropriate, which agreed with a previous report [20]. Moreover, the weak positive charge from the amino group dramatically encouraged the sorption of PAR on PUF.

### 3.3. Single Standard Calibration

In preliminary study, it was observed that the G/B value was directly proportional to the loading volume of a Pb^2+^ solution of a given concentration. This indicated that the single standard method could possibly be applied, as described earlier (in Section 2.2), when calculating the expected amount of Pb^2+^ (in microgram) of a solution percolating through a monolithic PUF–PAR rod. Various loading volumes from 5 to 100 mL of Pb^2+^ solutions at concentrations of 0.1 to 2 µg mL^−1^ were studied. The range of Pb^2+^ amounts at 10–40 μg was obtained using different volumes and concentrations of Pb^2+^ (Table 3). In addition, though the same amount of Pb^2+^ was obtained from diverse conditions, the same value of the resulting G/B signals was observed. The G/B value increased with the amount of Pb^2+^. However, at the higher amount of Pb^2+^ (30–40 μg), the G/B value became constant. This is due to the limitations of the PUF–PAR surface area and the amount of PAR.

The results of the studies confirm the possibility of employing the single standard calibration approach.

The lowest amount of Pb^2+^ in the linear calibration (Section 2.2) was 10 µg Pb^2+^. Considering a loading volume of 300 mL for 10 µg Pb^2+^, which was the last point of the calibration, would result in 33 µg L^−1^ Pb^2+^, which indicated the limit of quantitation (LOQ). The LOQ of the proposed procedure is lower than Thailand’s guidelines for the maximum allowance of lead, being 50 µg L^−1^ Pb^2+^.

It should be noted that, for an additional advantage when using the single standard approach for a given set of conditions (constant flow rate), if loading a sample solution with a given loading volume produced a G/B value lower than the lowest point of the linear calibration, the sample solution could be reloaded with a more appropriate loading volume to produce a G/B value within the linear range. Similarly, if loading a sample solution resulted in a higher G/B value, a lower reloading volume would provide a G/B value within the linear range. It is worth mentioning that the previous reports [9,12,13,14], employing sensors with electrochemical analysis of 3–17 min duration, provided reported LODs of 1–4.4 µg L^−1^ being converted to LOQs of 3–15 µg L^−1^, by calculating LOQ = 3.3LOD. The sensors with nanomaterials [8,10,11,55] reported LODs of 7.7−20 µg L^−1^ Pb^2+^, with an analysis time of 5−15 min. With the resin, AV-17 using PAR [17,19], LODs were reported to be 10−20 µg L^−1^ Pb^2+^, with an analysis time of 5 min. The proposed procedure in this work may be not as sensitive as those [9,12,13,14], but it is still useful for monitoring screening, in accordance with the guideline of 50 µg L^−1^ [1]. In addition, the proposed procedure offers other advantages, namely greater cost effectiveness when considering the sensor material (PUF–PAR), and simple operation with simple apparatus. The use of a smartphone offers the novel detection of lead via the developed chemical sensor (monolithic PUF–PAR rod), in association with other benefits, such as providing sampling locations for the possible mapping of the monitoring scheme. In terms of analysis time, the proposed procedure in this work requires a long period in order for the loaded monolithic PUF–PAR rod to be dried; parallel operations could be arranged to compensate for the time.

The proposed procedure indicates that its sensitivity would be useful for the on-site screening of drinking water in Thailand, which should be useful to some organizations such as the Pollution Control Department of Thailand, a rural waterworks authority (such as in Klity village, Kanchanaburi, Thailand), and for rural places without external power and where the budget is limited.

### 3.4. Interference Study

Using PAR as the chelating color agent, some co-existing metal ions in drinking water such as ${\mathrm{Ca}}^{2+}$, ${\mathrm{Fe}}^{3+}$,${\;\mathrm{Cu}}^{2+}$, and$\;{\mathrm{Zn}}^{2+}\;$were examined for potential interference. A higher standard deviation than ±15% of the G/B value was taken as interference. In this work, the ratios of [Ion]/[Pb^2+^] were evaluated, indicating the tolerant limits as 500 for ${\mathrm{Ca}}^{2+}$; 0.2 for ${\mathrm{Fe}}^{2+}$,$\;{\mathrm{Co}}^{2+}$,${\;\mathrm{Ni}}^{2+}$, and ${\mathrm{Cu}}^{2+}$; and 1 for ${\mathrm{Fe}}^{3+}$,${\;\mathrm{Zn}}^{2+}$, and ${\mathrm{Cd}}^{2+}$. The observed results may possibly be in connection with the potential for complexation formation of the metal ions with PAR, by considering the stability constants [56,57,58,59]. In a previous study [60], it was reported that the ratio of [Ion]/[Pb^2+^] was 500 for ${\mathrm{Na}}^{+}$, $K^{+}$, ${\mathrm{Cl}}^{-}$, ${\mathrm{CO}}_{3}^{2-}$, and ${\mathrm{NO}}_{3}^{-}$.

## 4. Materials and Methods

### 4.1. Apparatus

Digital images of Pb^2+^–PAR complexes on monolithic PUF–PAR rods were taken using a smartphone (Lumia 930, Nokia, Tampere, Finland) in manual mode. The smartphone camera settings were as follows: white balance, daylight; ISO, 200; shutter speed, 1:3200; brightness, 0.5; and zoom, 20×. A light-controlled photograph box (UDIOBOX UDIO BIZ 40 × 40 × 40 cm, Bangkok, Thailand) was used. ImageJ software (National Institutes of Health, Bethesda, ML, USA) was chosen for processing the images. An inductively coupled plasma emission spectrometer, ICP-OES (Perkin Elmer, Optima 8000, Waltham, MA, USA), was used for method validation. A digital pH meter (METTLER TOLEDO, Greifensee, Switzerland) was employed for measuring the pH of solutions.

### 4.2. Reagents and Materials

All chemical reagents used in this work were of analytical grade. Deionized water was used for solution preparation.

A 1000 µg mL^−1^ stock solution of Pb^2+^ was prepared by dissolving 0.160× *g* of Pb(NO_3_)_2_ (Loba Chemie, Mumbai, India) in 100 mL of water in a volumetric flask. Working solutions with various concentrations of Pb^2+^ were prepared daily by diluting with deionized water.

The solution of 0.05% *w*/*v* of 4-(2-pyridylazo) resorcinol (PAR) at pH 7 was prepared by dissolving 0.05 g of 4-(2-pyridylazo) resorcinol (TCI, Tokyo, Japan) in 1 mol L^−1^ NaOH, adjusting the pH to 7 with 2 mol L^−1^ of nitric acid, and diluting it to 100 mL in a volumetric flask with deionized water.

### 4.3. Preparation of Monolithic PUF–PAR Rod

The monolithic PUF rod was synthesized by mixing methylene diphenyl diisocyanate (MDI; IRPC, Rayong, Thailand) with polyol (polyether; IRPC, Rayong, Thailand) at a ratio of 2:1 with a few drops of water and silicone oil in a beaker. Two open-ended glass tubes, each 0.8 cm i.d. × 1.0 cm o.d. × 5.0 cm in length, were plugged into the mixture. The reaction was allowed to continue for 5 min. The obtained PUF rod was approximately 2 cm in height. The synthesized PUF rods were then cleaned with water and loaded with 0.05% *w*/*v* PAR (pH 7) for 1 h. Finally, the loaded foam was rinsed with water to remove the excess PAR before drying at room temperature overnight. The monolithic PUF–PAR rods could be used for at least a week.

### 4.4. General Procedure for Lead Determination Using Monolithic PUF–PAR Rod

The monolithic PUF–PAR rod was loaded by an analyte solution with a desired volume. Image processing was performed via ImageJ to obtain a color value. A single standard calibration was plotted in terms of µg Pb^2+^ vs. the signal of an image property. The amount of Pb^2+^ in the sample was then calculated.

## 5. Conclusions

A novel method for monitoring lead contents in water, by using smartphone detection and employing PUF immobilized with a PAR monolithic rod as a ready-to-use simple chemical sensor was proposed, by following the IUPAC definition of a chemical sensor. The PUF could be easily synthesized in simple lab conditions and lead to the simple fabrication of a monolithic PUF rod. With a PAR solution being loaded to the rod, the obtained monolithic PUF–PAR rod, serving as a ready-to-use chemical sensor, has a shelf-life of at least one week. The monolithic characteristics offer advantages over the previous conventional forms of PUF, including the smooth flow of the loading of an analyte solution (standard/sample), and the fact that no sample pretreatment is required. Lead content in a water sample can be assayed via the single standard calibration approach, with the concept of a one-shot image, in one single operation with nine rods: six rods for standards of different amounts of lead loaded on the monolithic PUF–PAR rods, and three rods of a sample for triplicate analysis. By this method, the triplicate results of the final concentration of lead in the sample can be obtained even when using different loading volumes. This offers a method through which to verify the obtained results. The additional advantage is that if the operation results in the loaded amounts of Pb^2+^ producing a color, with PAR being outside the calibration graph, which is a plot of µg Pb^2+^ loaded vs. the G/B value, then a new operation can be re-run immediately so that an appropriate result can be obtained. The developed method offers various benefits, even with the simple apparatus used. It is useful for screening following Thailand’s guidelines for health effects, which list a maximum allowance of 50 µg L^−1^ for lead in drinking water. The smartphone serves not only as a detector, but also as the provider of the sampling location. This leads to the ability to map lead in water as part of a cost-effective schedule.

## Figures and Tables

**Table 1:** Assay using the proposed procedure for one-shot image of a sample with triplicate results via a single standard calibration approach.

Position (in Figure 2)	Loading Pb^2+^			Intensity		G/B value ^c^	Pb^2+^ found	
	µg	µg mL^−1^	Loading volume(mL)	G value	B value		µg ^a^	Concentration (µg mL^−1^)
A	10	0.2	50	200	80	2.5	−	−
D	10	0.5	20	200	80	2.5	−	−
B	20	0.1	200	200	100	2.0	−	−
C	20	0.1	200	200	90	2.2	−	−
E	30	0.3	100	170	95	1.8	−	−
F	30	0.5	60	170	95	1.8	−	−
I	−	−	40	200	90	2.2	18	0.4 ^b^
H	−	−	50	195	105	1.9	27	0.5 ^b^
G	−	−	70	200	115	1.7	32	0.4 ^b^

^a^ µg from calibration; ^b^ see text; ^c^ the ratio of the G/B value, where the G (green) value and B (blue) value refer to the color intensities of the color mode.

**Table 2:** Assay of lead contents in drinking water samples from different districts in Bangkok.

Sample	Monitoring site ^a^	Added Pb^2+^ std (µg L^−1^)	Found Pb^2+^				t_observed_ ^d^
			Proposed method (*n* = 3)			ICP-OES (*n* = 3)	
			µg ^c^	µg L^−1^	% Recovery	µg L^−1^	
S1 (Dindaeng)	13° 46′ 12.80644″ N, 100° 33′ 31.986″ E	−	ND	−	−	ND	2.70
		50 ^b^	18 ± 2	60 ± 7	120	49 ± 0.4	
S2 (Bangkapi)	13° 46′ 7.2408″ N, 100° 38′ 30.03″ E	−	ND	−	−	ND	1.22
		50 ^b^	18 ± 5	60 ± 17	120	48 ± 0.4	
S3 (Huai Khwang)	13° 48′ 1.1916″ N, 100° 35′ 1.41″ E	−	ND	−	−	ND	0.79
		50 ^b^	15 ± 4	50 ± 13	100	44 ± 0.8	
S4 (Thungkru)	13° 38′ 58.182″ N, 100° 29′ 46.9896″ E	−	ND	−	−	ND	2.07
		50 ^b^	18 ± 3	60 ± 10	120	48 ± 0.4	

^a^ monitoring sites with latitude and longitude; ^b^ a total of 50 Pb^2+^ std (µg L^−1^) was purposely added following the maximum acceptable concentration of lead in drinking water [1]; ^c^ µg evaluated from the calibration; ND = not detectable; ^d^ t_observed_ values were less than theoretical values (2.92 at 95% confidence level), indicating no significant difference.

**Table 3:** Loading Pb^2+^ solutions of different concentrations and volumes for single standard calibration (*n* = 3).

Amount of Pb^2+^ (μg)	Concentration of Pb^2+^ (μg mL^−1^)	Loading Volume (mL)	G/B ^a^ ± SD ^b^
10	0.1	100	2.5 ± 0.3
10	0.2	50	2.5 ± 0.3
10	2.0	5	2.6 ± 0.2
20	0.2	100	2.1 ± 0.2
20	0.5	40	2.1 ± 0.3
20	2.0	10	2.0 ± 0.1
30	0.3	100	1.7 ± 0.1
30	0.5	60	1.8 ± 0.1
30	2.0	15	1.8 ± 0.1
40	0.5	80	1.6 ± 0.2
40	2.0	20	1.6 ± 0.1

^a^ The ratio of G/B value, where the G (green) value and B (blue) value refer to the color intensities of the color mode. ^b^ ± SD is the standard deviation of triplicate measurements of the G/B values.

## Data Availability

All of the data are reported in this manuscript and supplementary material.

## Supplementary Materials

Figure S1: Lead assay with smartphone detection: A = ready-to-use sensor, B = detection (loading and photographing) 18 and C = evaluation.
