# Supplementary material for: Lead Assays with Smartphone Detection Using a Monolithic Rod with 4-(2-Pyridylazo) Resorcinol
Source: Molecules. 2021 Sep 21;26(18):5720. doi: 10.3390/molecules26185720 (PMC8466971; doi:10.3390/molecules26185720)
Supplement: Supplementary file 1 [file molecules-26-05720-s001.zip › molecules-1366151-supplementary.pdf]

*molecules*

*Article*

# Lead Assays with Smartphone Detection Using a Monolithic Rod with 4-(2-Pyridylazo) Resorcinol

**Piyanat Issarangkura Na Ayutthaya [1], Chonnipa Yeerum [1], Kullapon Kesonkan [1], Kanokwan Kiwfo [2], Kate Grudpan [2,*] Norio Teshima [3], Hiroya Murakami [3] and Monnapat Vongboot [1,*]**

[1] Department of Chemistry, Faculty of Science, King Mongkut's University of Technology Thonburi, Bangkok, 10140, Thailand; piyanat.tp@gmail.com (P.I.N.A.); chonnipa.yeerum@gmail.com (C.Y.); kullapon.kesonkan@gmail.com (K.K.)

[2] Center of Excellence for Innovation in Analytical Science and Technology and Department of Chemistry, Faculty of Sciences, Chiang Mai University, Chiang Mai, 50200, Thailand; k.kanokwan11@gmail.com

[3] Department of Applied Chemistry, Aichi Institute of Technology, 1247 Yachigusa, Yakusa-cho, Toyota 470-0392, Japan; teshima@aitech.ac.jp (N.T.); hmurakami@aitech.ac.jp (H.M.)

* Correspondence: sumalee.tan@kmutt.ac.th (M.V.); Tel.: +662-470-8969, kgrudpan@gmail.com (K.G.); Tel.: +66-5394-1917

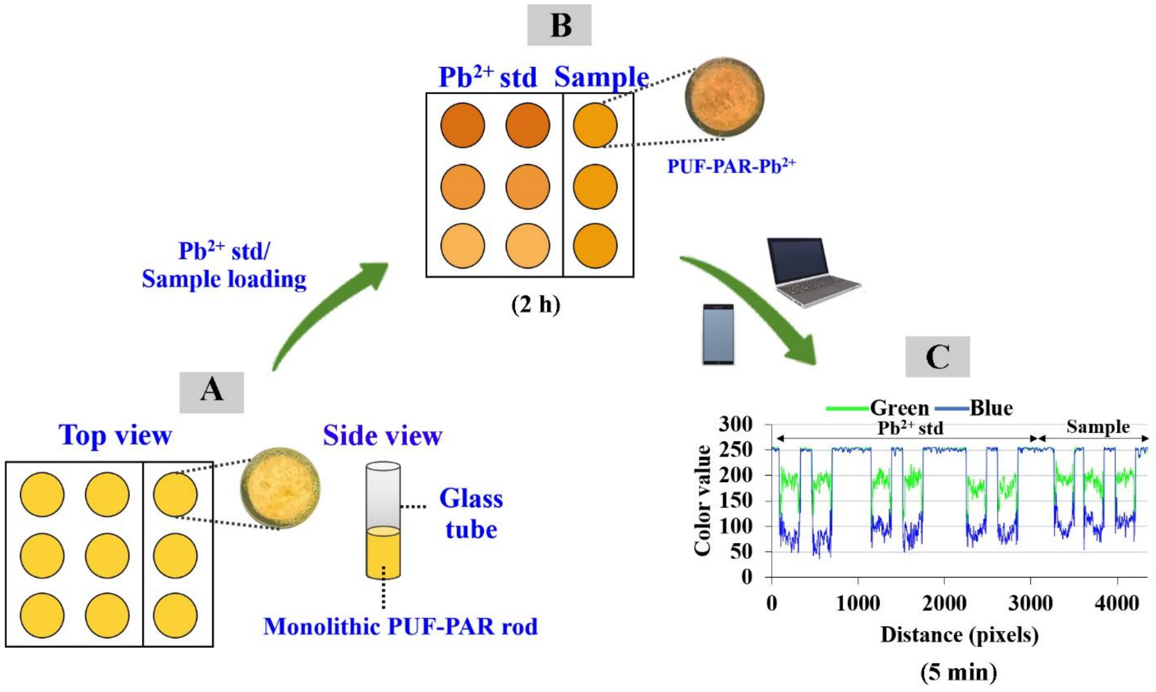


**Figure S1.** Lead assay with smartphone detection: A = ready-to-use sensor, B = detection (loading and photographing) and C = evaluation

Figure S1 illustrates lead assay with smartphone detection, including three steps of the ready-to-use sensor, detection (loading and photographing), and evaluation.
